# The Effect of Changes in the Body Mass Index After Total Knee and Hip Arthroplasty on Patient Functional Scores

**DOI:** 10.7759/cureus.71790

**Published:** 2024-10-18

**Authors:** Cumhur Deniz Davulcu, Mete Ozer

**Affiliations:** 1 Department of Orthopaedics and Traumatology, Istanbul University-Cerrahpasa, Cerrahpasa Faculty of Medicine, Istanbul, TUR

**Keywords:** body mass index, functional scores, obesity, postoperative weight change, total hip arthroplasty, total knee arthroplasty, weight loss

## Abstract

Introduction

High body mass index (BMI) often causes immobility and functional impairment before arthroplasty. Patients expect weight loss post-surgery due to increased mobility, but paradoxically, studies show that weight may not decrease and might even increase postoperatively. This study aims to evaluate if patients lose weight after total knee arthroplasty (TKA) and total hip arthroplasty (THA) and to examine the impact of BMI change on functional scores.

Methods

We analyzed 459 patients who underwent primary TKA and THA between January 2018 and December 2022, with a two-year follow-up. Patients with incomplete data or bariatric surgery were excluded. Demographic characteristics, BMI, Oxford scores, comorbidities, corticosteroid use, physiotherapy, and surgery type (unilateral or bilateral) were assessed.

Results

Patients showed a significant increase in BMI postoperatively, especially those with comorbidities and younger age. Throughout the entire follow-up period, the BMI value of the knee group was higher than that of the hip group. Comorbidities significantly influenced BMI increase, while corticosteroid use and physiotherapy follow-up did not. Oxford scores improved postoperatively, but the presence of comorbidities and physiotherapy follow-up negatively impacted score changes. A negative correlation between BMI change and Oxford score change was observed (p = 0.013), indicating that increased BMI is associated with less improvement in functional scores. This correlation was significant for hip arthroplasty patients (p = 0.000), but not for knee arthroplasty patients (p = 0.822).

Conclusions

BMI changes post-TKA and THA are influenced by various clinical and demographic factors. Increased BMI negatively affects functional outcomes, particularly in hip arthroplasty patients. Comorbidities significantly influenced weight gain, while oral corticosteroid use had no notable effect on BMI. Patients undergoing bilateral procedures experienced lower BMI increases. The hip group gained more weight, but the knee group's BMI remained higher throughout the follow-up. In terms of Oxford scores, comorbidities, corticosteroid use, affected joints, and age did not significantly impact outcomes. Patients under physiotherapist supervision showed smaller score increases. While bilateral procedure patients achieved higher scores, simultaneous bilateral procedures did not lead to greater increases. These findings highlight the importance of weight control and rehabilitation in improving postoperative recovery and quality of life.

## Introduction

High body mass index (BMI) is reported by patients as a cause of immobility and functional impairment in the preoperative period of arthroplasty. Patients claim that mobility restrictions due to joint pain negatively impact weight control. Patients awaiting knee and hip arthroplasty expect a decrease in weight following surgery due to increased mobility. Paradoxically, studies have shown that patients cannot lose weight in the postoperative period and may even gain weight, despite the absence of statistically significant differences [1-5]. Weight changes after knee and hip arthroplasties are generally minimal, but factors like gender, age, and genetics can lead to differing weight change patterns among groups [3]. Following knee and hip arthroplasty, an increase in daily step counts and activity levels has been observed in association with the alleviation of pain complaints [6]. It is known that the increase in physical activity has an impact on weight control in obesity treatment [7]. In light of this information, it can be assumed that an increase in activity after joint arthroplasty would be an expected outcome, along with improved weight control.

Comorbidities such as type 2 diabetes mellitus, primary hypertension, thyroid autoimmunity, coronary artery disease, rheumatoid arthritis, and gout, which are also found in arthritis patients, are comorbidities associated with obesity [8-12]. The presence of multiple comorbidities leads to lower health utility values in osteoarthritis patients [13]. The presence and multiplicity of these comorbidities have not been clearly defined in relation to BMI following arthroplasty surgery. Patients undergoing arthroplasty treatment may require corticosteroid use due to rheumatic diseases or other conditions requiring corticosteroid therapy. These patients may need to continue corticosteroid treatment after surgery. It is known that corticosteroids have effects that can lead to weight gain [14]. Postoperative physical therapy is a recommended treatment after joint arthroplasty [15]; however, its effectiveness in the long term has not been conclusively established [16,17]. We expect a decrease in BMI levels with the promotion of physical therapy and increased activity levels. However, we have not come across an examination of the relationship between physical therapy and BMI change. Understanding the factors affecting weight changes after joint arthroplasty is crucial, especially in obese patients. This knowledge helps orthopedic surgeons guide patients in managing weight effectively post-surgery.

The main objectives of this study are to assess whether patients experience weight loss following total knee arthroplasty (TKA) and total hip arthroplasty (THA) and to evaluate the influence of BMI variations on functional outcomes. Additionally, the study aims to examine the impact of comorbidities, oral corticosteroid use, postoperative physiotherapy treatment, and the distinction between unilateral and bilateral surgery on weight fluctuations. These objectives are intended to enhance understanding of the effects of surgical procedures on weight changes during follow-up.

## Materials and methods

This study was conducted at Istanbul University-Cerrahpasa, Istanbul, and approved by the Istanbul University-Cerrahpasa Clinical Research Ethics Committee (Approval No. Y7MQG7gO). Informed consent was obtained from all patients prior to participation. The inclusion criteria for the study were that participants had undergone total knee or total hip arthroplasty due to an arthritis diagnosis (either primary or secondary), had a minimum follow-up period of two years, and had recorded Oxford scores during both the preoperative period and follow-up. Additionally, BMI (kg/m²) values needed to be documented during the same periods. Information on comorbidities, corticosteroid use, physiotherapy follow-up, surgical side, and whether the procedure was simultaneous or staged also had to be recorded and accessible. The exclusion criteria included having undergone surgical interventions that could affect BMI changes (such as bariatric surgery), having a diagnosis of malignancy, having had revision arthroplasties, undergoing arthroplasty for non-arthritis indications (such as trauma or tumors), and having incomplete two-year follow-up records (including BMI values, Oxford scores, comorbidities, corticosteroid use, physiotherapy follow-up, surgical side, and simultaneous or staged procedure information).

A total of 1,166 patients were initially screened, of which 459 met the inclusion criteria for analysis. The study encompassed patients treated between January 2018 and August 2022. Surgical techniques included THA via a posterior approach for the hip group and TKA via a medial parapatellar approach for the knee group. Their demographic characteristics were examined, and patients were followed postoperatively at six, 12, and 24 months. The evaluation included BMI (kg/m²) and functional scores. Height and weight measurements were taken using a stadiometer with a mechanical patient weighing scale, which is available in our clinic. Oxford score forms were completed by the patients upon admission on the preoperative day and during follow-up visits, and the forms were received on the same day. Comorbidities recorded included diabetes mellitus, hypertension, cardiac disease, pulmonary disease, neurological disorders, rheumatoid disease, chronic renal failure, thyroid disease, and liver disease. The use of ongoing oral corticosteroids related to existing conditions was assessed. It was assessed whether patients received physiotherapy sessions under the supervision of a physiatrist. Patients were categorized into unilateral and simultaneous bilateral treatment groups. The Oxford hip score was utilized for the hip cohort, while the Oxford knee score was employed for the knee cohort as the clinical scoring system. The Oxford hip score and Oxford knee score are measures for assessing pain and function in patients undergoing lower limb arthroplasty. Each joint-specific score consists of 12 questions, answered on a five-point response scale, with total scores ranging from 0 to 48, where higher scores indicate better functional ability. Using the same scoring system for both knee and hip allows for a unified and comparative assessment of the two surgical procedures.

Statistical analysis

Descriptive statistics outlined demographic and clinical characteristics. Repeated measures ANOVA assessed changes in BMI and Oxford scores, with attention to the fulfillment of assumptions such as normality and sphericity. Missing data were managed using [insert method, e.g., imputation, exclusion] to ensure robust results. Adjustments for potential confounding variables, such as comorbidities and corticosteroid use, were made where applicable. Pearson correlation examined relationships between changes in BMI and Oxford scores. Independent t-tests and ANOVA compared BMI and Oxford scores among different groups, ensuring that assumptions for these tests were satisfied. A significance level (p-value) of 0.05 was used. The statistical analysis was conducted using IBM SPSS Statistics for Windows, Version 24 (Released 2016; IBM Corp., Armonk, New York, United States).

## Results

The characteristics of the groups are shown in Table 1.

**Table 1:** Various clinical and demographic characteristics of the patients Data are expressed as percentages (%), with sample size indicated as (n). Statistical significance was considered at p < 0.05.

Variables	Patients status	n	%
Presence of comorbidities	None	144	31.4
	Single comorbidity	171	37.3
	Two or more comorbidities	144	31.4
Oral corticosteroid use	None	402	88.4
	Yes	53	11.6
Postoperative physiatrist follow-up	None	285	62.5
	Yes	171	37.5
Side (including older surgeries)	Right	179	39.1
	Left	159	34.7
	Bilateral	120	26.2
Unilateral/bilateral	Unilateral	308	67.5
	Bilateral	148	32.5
Type of arthroplasty	Knee	194	42.3
	Hip	265	57.7
Age group	Under 35 years	48	10.8
	35-45 years	50	11.2
	46-55 years	93	20.9
	56-65 years	112	25.2
	Over 65 years	142	31.9

In Table 2, a statistically significant change has been observed in preoperative and postoperative BMI values, indicating weight gain in patients after knee and hip arthroplasty.

**Table 2:** Preoperative and postoperative BMI (kg/m²) changes for all patients Data are presented as mean ± standard deviation (X ± SD) and median (M), with sample size indicated as (n). Statistical significance was considered at p < 0.05. BMI: body mass index

BMI calculation times	n	Minimum	Maximum	X	sd	M
Preoperative BMI	459	16.84	49.00	28.76	4.85	28.84
BMI at 6 months	459	18.73	44.00	28.98	4.81	29.00
BMI at 12 months	459	18.34	46.00	29.18	4.78	29.00
BMI at 24 months	459	18.00	46.00	29.39	4.62	29.37

**Figure 1:**
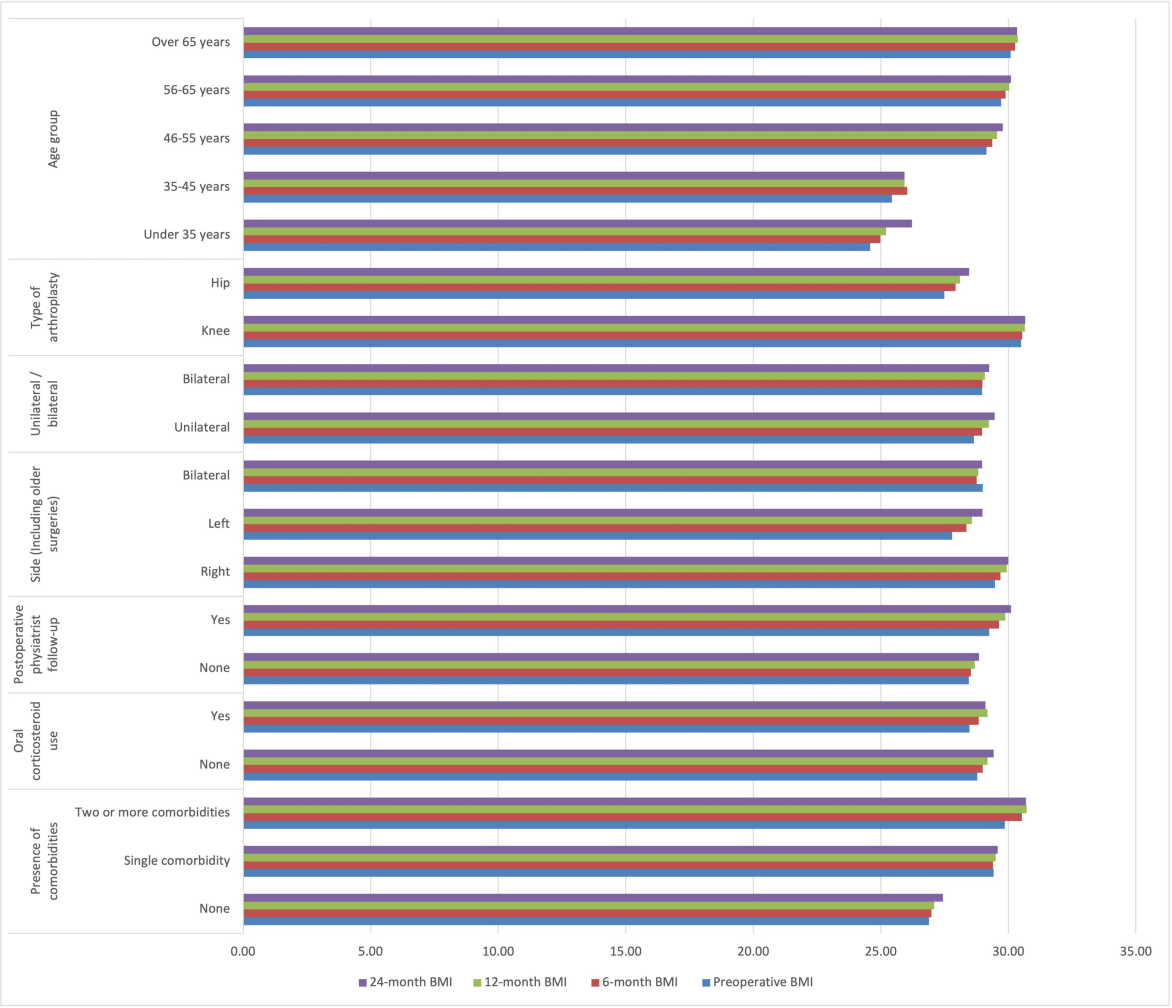
BMI (kg/m²) changes and variables BMI: body mass index

From the factors shown in Table 3, the presence of comorbidities has been associated with postoperative BMI increase. Oral corticosteroid usage did not show a significant interaction with BMI change. Postoperative physiatrist follow-up did not show a significant interaction with BMI change, but it approached significance, indicating that patients under physiatrist follow-up tended to gain relatively more weight. The side of surgery showed a significant interaction with BMI change. The unilateral or bilateral surgery status also showed a significant interaction with BMI change. A significant interaction was observed between knee and hip arthroplasty, with the hip group experiencing a greater numeric weight gain, while the BMI of the knee group remained higher than that of the hip group throughout the entire follow-up period. Among age groups, there was no significant interaction in BMI change, but relatively younger patients were observed to gain more weight (Figure 1).

**Table 3:** BMI (kg/m²) changes and variables Data are presented as mean ± standard deviation (X ± SD). Statistical significance was considered at p < 0.05. BMI: body mass index

Variables	Patients status	Preoperative BMI		6-month BMI		12-month BMI		24-month BMI	
		X	sd	X	sd	X	sd	X	sd
Presence of comorbidities	None	26.88	4.76	26.97	4.60	27.08	4.63	27.44	4.48
	Single comorbidity	29.41	4.67	29.39	4.48	29.50	4.39	29.58	4.27
	Two or more comorbidities	29.85	4.64	30.52	4.71	30.71	4.68	30.69	4.62
Oral corticosteroid use	None	28.78	4.72	29.00	4.68	29.18	4.61	29.41	4.43
	Yes	28.47	4.88	28.82	5.19	29.17	5.19	29.09	5.18
Postoperative physiatrist follow-up	None	28.45	4.80	28.52	4.71	28.68	4.71	28.84	4.55
	Yes	29.24	4.94	29.63	4.85	29.87	4.76	30.09	4.59
Side (including older surgeries)	Right	29.46	4.79	29.69	4.72	29.93	4.56	30.00	4.41
	Left	27.79	4.58	28.36	4.76	28.56	4.82	28.98	4.78
	Bilateral	28.99	5.14	28.75	4.93	28.82	4.96	28.97	4.72
Unilateral/bilateral	Unilateral	28.64	4.75	28.96	4.80	29.23	4.72	29.46	4.58
	Bilateral	28.97	5.11	28.98	4.88	29.07	4.96	29.24	4.76
Type of arthroplasty	Knee	30.49	4.52	30.54	4.42	30.64	4.33	30.65	4.24
	Hip	27.49	4.70	27.92	4.78	28.10	4.82	28.46	4.68
Age group	Under 35 years	24.57	4.70	24.98	4.45	25.20	4.47	26.21	4.27
	35-45 years	25.43	3.69	26.03	3.84	25.93	4.04	25.92	4.03
	46-55 years	29.14	5.31	29.36	5.17	29.55	5.29	29.77	5.28
	56-65 years	29.71	4.33	29.89	4.26	30.04	4.26	30.10	4.11
	Over 65 years	30.09	4.07	30.26	4.25	30.36	4.06	30.34	4.01

Table 4 shows that patients gained weight after surgery, as indicated by a statistically significant difference in pre-and postoperative BMI values. Comorbidity was identified as a significant factor in weight gain. The side of surgery (unilateral or bilateral) and the type of joint (knee or hip) were also significant factors affecting changes in BMI. Patients who underwent simultaneous bilateral procedures had lower BMI values and experienced less BMI increase. A significant interaction was observed between knee and hip arthroplasty, with the hip group showing greater numeric weight gain, while the BMI of the knee group remained higher than that of the hip group throughout the entire follow-up period. Oral corticosteroid use and physical therapy follow-up did not significantly affect BMI changes, and there was no significant difference in BMI change between age groups. In conclusion, Table 4 indicates that postoperative BMI changes are influenced by factors such as comorbidity, surgical side, and type of surgery.

**Table 4:** Examination of BMI (kg/m²) changes with repeated measures ANOVA Data are presented as mean ± standard deviation (X ± SD). Variables include body mass index (BMI), group test (GT), standard deviation (SD), coefficient (CO), F-statistic (F), and p-value (p). Statistical significance was considered at p < 0.05.

BMI and related factors	Type III GT	sd	CO	F	p	Partial eta squared
BMI	10.911	1.711	6.375	8.056	0.001	0.024
BMI * Comorbidities	16.099	3.423	4.703	5.944	0.000	0.035
BMI * Steroid use	2.747	1.711	1.605	2.029	0.140	0.006
BMI * Postoperative physiatrist follow-up	4.203	1.711	2.456	3.104	0.054	0.009
BMI * Side	16.573	3.423	4.842	6.119	0.000	0.036
BMI * Unilateral or bilateral	6.030	1.711	3.523	4.452	0.016	0.013
BMI * Knee or hip arthroplasty	7.956	1.711	4.649	5.875	0.005	0.018
BMI * Age group	6.748	6.846	0.986	1.246	0.277	0.015

In conclusion, these findings indicate that BMI changes are influenced by various clinical and demographic factors.

As shown in Table 5, statistically significant increases were observed in the preoperative and postoperative Oxford score values. The presence of comorbidities did not show a significant interaction with postoperative score change. Oral corticosteroid use did not show a significant interaction with score change. Postoperative physiatrist follow-up was paradoxically associated with a decrease in score change. The side of surgery showed a significant interaction with score change. Unilateral or bilateral surgery also showed a significant interaction with score change, with higher scores reported in the bilateral group. There was no significant interaction between knee or hip arthroplasty. Among age groups, there was no significant interaction in score change. 

**Table 5:** Oxford scores, including Oxford knee score and Oxford hip score, were utilized for all patients Data are presented as mean ± standard deviation (X ± SD) and median (M), with sample size indicated as (n). Statistical significance was considered at p < 0.05.

Oxford score calculation times	n	Minimum	Maximum	X	sd	M
Preoperative Oxford score	459	6.00	42.00	18.41	5.28	18.00
Oxford score at 6 months	459	7.00	48.00	33.13	7.43	33.00
Oxford score at 12 months	459	11.00	48.00	34.91	7.26	34.00
Oxford score at 24 months	459	12.00	49.00	35.97	7.24	36.00

**Figure 2:**
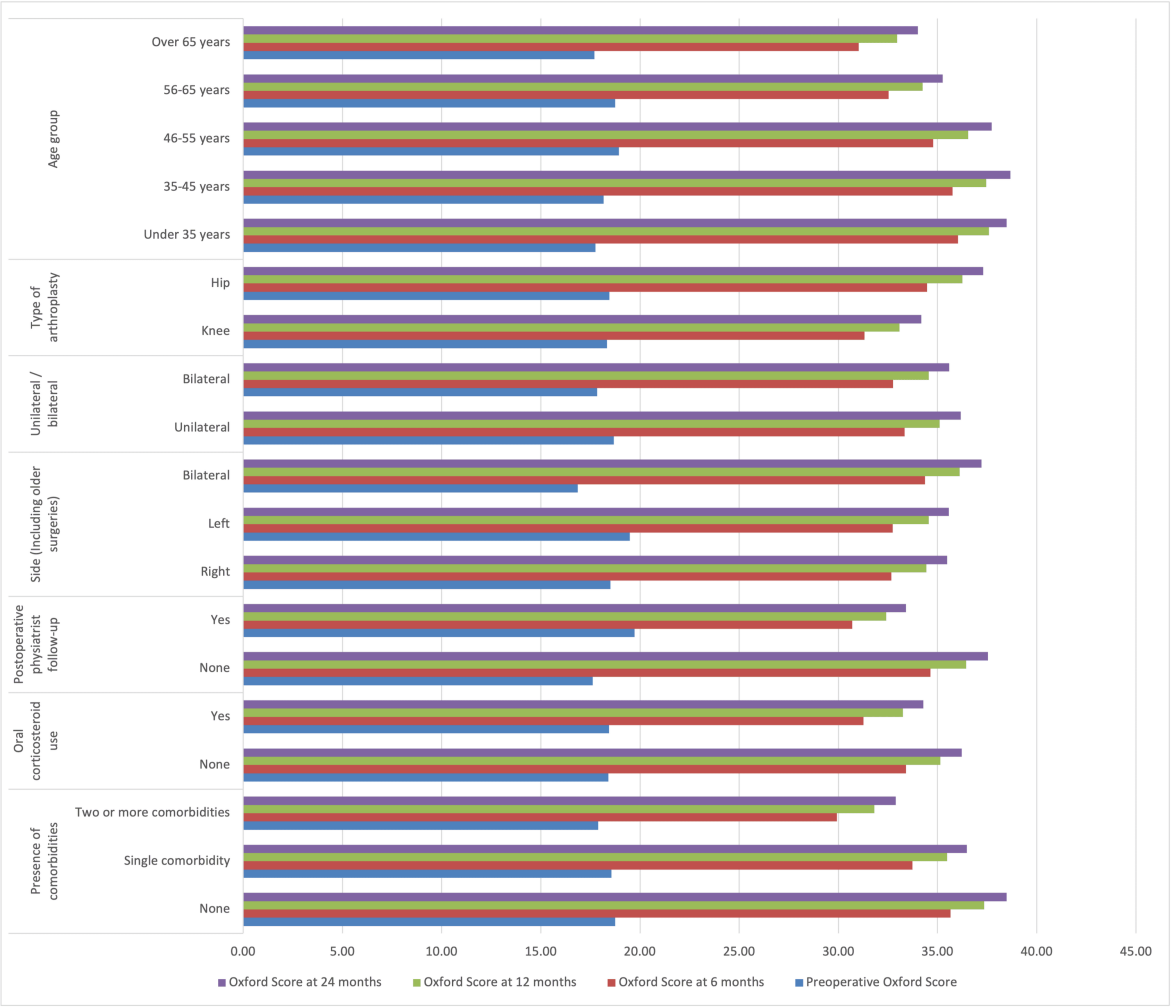
Oxford scores changes and variables

As shown in Table 6, the Oxford score increased across all groups during the follow-up period. The number of comorbidities, corticosteroid use, affected joints, and age group did not have a significant impact on the scores. However, physiotherapist supervision and the side of surgery did influence the scores. (Figure 2) The results of the repeated ANOVA analysis conducted to examine the effects of these variables on the groups are presented in Table 7.

**Table 6:** Oxford scores changes and variables Data are presented as mean ± standard deviation (X ± SD). Statistical significance was considered at p < 0.05.

Variables	Patients status	Preoperative Oxford score		6-month Oxford score		12-month Oxford score		24-month Oxford score	
		X	sd	X	sd	X	sd	X	sd
Presence of comorbidities	None	18.75	5.69	35.66	7.09	37.34	6.85	38.48	6.88
	Single comorbidity	18.57	4.99	33.73	6.85	35.48	6.73	36.47	6.71
	Two or more comorbidities	17.89	5.20	29.92	7.30	31.81	7.20	32.89	7.12
Oral corticosteroid use	None	18.41	5.31	33.40	7.33	35.14	7.11	36.21	7.09
	Yes	18.43	5.16	31.26	8.01	33.25	8.13	34.28	8.09
Postoperative physiatrist follow-up	None	17.61	5.48	34.63	7.51	36.44	7.24	37.54	7.22
	Yes	19.72	4.69	30.69	6.67	32.42	6.62	33.40	6.56
Side (including older surgeries)	Right	18.50	4.39	32.67	7.29	34.43	7.17	35.48	7.10
	Left	19.48	5.60	32.74	6.64	34.55	6.52	35.57	6.77
	Bilateral	16.87	5.74	34.36	8.49	36.12	8.20	37.22	7.95
Unilateral/bilateral	Unilateral	18.69	5.10	33.34	6.93	35.10	6.82	36.17	6.87
	Bilateral	17.84	5.64	32.75	8.43	34.55	8.16	35.58	8.02
Type of arthroplasty	Knee	18.34	4.37	31.31	6.04	33.08	5.90	34.18	5.87
	Hip	18.46	5.87	34.47	8.05	36.25	7.85	37.29	7.85
Age group	Under 35 years	17.75	6.10	36.02	9.13	37.58	8.68	38.48	8.53
	35-45 years	18.16	5.00	35.76	8.07	37.45	8.06	38.67	7.96
	46-55 years	18.94	5.08	34.77	6.87	36.55	6.68	37.72	6.76
	56-65 years	18.75	5.26	32.54	6.67	34.25	6.65	35.25	6.60
	Over 65 years	17.70	5.17	31.03	6.93	32.96	6.71	34.01	6.70

**Table 7:** Examination of Oxford score change with repeated measures ANOVA Data are presented as mean ± standard deviation (X ± SD). Variables include degrees of freedom (df), F-statistic (F), and p-value (p). Statistical significance was considered at p < 0.05.

Oxford scores and related factors	Type III sum of squares	df	Mean square	F	p	Partial sta squared
Oxford	24313.296	1.069	22740.978	440.733	0.000	0.511
Oxford * Comorbidities	294.873	2.138	137.902	2.673	0.066	0.013
Oxford * Steroid use	21.540	1.069	20.147	0.390	0.546	0.001
Oxford * Postoperative physiatrist follow-up	1007.591	1.069	942.431	18.265	0.000	0.041
Oxford * Side	2603.439	2.138	1217.538	23.597	0.000	0.101
Oxford * Unilateral or bilateral	1631.637	1.069	1526.120	29.577	0.000	0.065
Oxford * Knee or hip arthroplasty	158.193	1.069	147.963	2.868	0.088	0.007
Oxford * Age group	295.705	4.277	69.146	1.340	0.252	0.013

As shown in Table 7, a repeated ANOVA analysis of changes in the Oxford score revealed an increase in postoperative Oxford score values. In this analysis, the presence of comorbidities, oral corticosteroid use, the involved joint, and age did not significantly affect the outcomes. Patients under physiotherapist supervision showed less increase in their scores. While patients who underwent bilateral procedures achieved higher Oxford scores, those who had simultaneous bilateral procedures did not demonstrate a greater increase in scores.

Overall, these findings indicate that Oxford score changes are influenced by various clinical and demographic factors.

Relationship between BMI change and Oxford score change

The central focus of this study is the relationship between BMI change and Oxford score change. As shown in Table 8, a negative correlation exists between BMI change and Oxford score change across all groups. This means as BMI increases, Oxford scores tend to decrease or improve less significantly.

**Table 8:** Correlation relationship between BMI (kg/m²) change and Oxford Score change. This analysis evaluates the relationship between BMI change and Oxford score change in the general population and specifically in the knee arthroplasty and hip arthroplasty groups. Data are presented as correlation coefficients (r) and p-values (p). Statistical significance was considered at p < 0.05. BMI: body mass index

Correlation relationship		Oxford change		
		In all groups (knee arthroplasty/hip arthroplasty)	Knee group	Hip group
BMI change	r	-0.131	0.018	-0.280
	p	0.013	0.822	0.000

Knee arthroplasty group

No significant correlation was found between BMI change and Oxford score change (p = 0.822), indicating BMI change does not affect Oxford scores in knee arthroplasty patients.

Hip arthroplasty group

A significant negative correlation was observed in the hip arthroplasty group (p = 0.000). This suggests that as BMI increases, Oxford scores decrease significantly, highlighting a negative impact of BMI gain on functional outcomes in hip arthroplasty patients.

## Discussion

Based on our data, BMI increased after knee and hip arthroplasty. In the hip arthroplasty group, this increase negatively impacted clinical scores, while no such effect was seen in the knee group. We also examined how age, comorbidities, oral corticosteroid use, postoperative physiatrist follow-up, and the type of procedure (unilateral or bilateral) influenced BMI and clinical scores.

Surgeons have been wary of performing joint replacements on obese patients due to concerns about complications and outcomes. However, studies show that obese patients can achieve similar clinical outcomes and complication rates as those with normal BMI. Additionally, joint replacement can improve activity levels and aid in managing obesity [18].

The most significant risk factor for weight gain in the postoperative period has been identified as obesity [19,20]. It has been shown that preoperative weight loss also leads to rapid weight gain after surgery [21].

Studies often report no change in weight after joint arthroplasty, with some patients even gaining weight. This suggests that factors beyond limited mobility due to osteoarthritis contribute to weight issues [1-5]. In our study, we examined how age, gender, race, sociocultural factors, comorbidities, oral corticosteroid use, postoperative physiatrist follow-up, and the type of arthroplasty (knee or hip, unilateral or bilateral) affect weight.

It has been suggested that weight gain following joint arthroplasty may pose potential risks to overall health [2]. It is believed that weight control not only leads to improvements in overall health but also has positive effects on implant survival [4].

Postoperative weight gain from joint replacement is partly due to implants and cement, which can be up to 364 grams heavier than natural tissue [22]. However, weight changes after surgery cannot be solely attributed to the implants.

Even with successful surgeries, the lack of expected BMI reduction suggests that obesity should be managed separately from arthritis-related mobility issues [23]. The noticeable increase in clinical scores despite the increase in BMI in our study supports this notion.

It has been shown that having a lower body weight is an effective factor for success and patient satisfaction after arthroplasty [24]. Therefore, surgeons aim for weight control in patients during the preoperative period. Including dietitian consultations in postoperative care has proven highly effective for weight control [25] and should be considered in post-arthroplasty follow-up.

Contrary to the view that weight loss has a positive impact on arthroplasty outcomes, it has been argued, through the investigation of a large cohort, that changes in BMI do not affect functional scores and prosthetic survival [26]. It has been shown that BMI does not change significantly; younger patients can lose weight, while older patients may struggle to do so. Additionally, weight loss tends to occur more in patients with higher BMI levels [26]. On the contrary, although there was no significant difference in our own study, relatively younger patients gained more weight. As in our study, it has also been argued that young patients have a higher risk of gaining weight after surgery [2,3]. Our study data reveals a negative correlation between BMI changes and clinical scores in patients undergoing TDA and TKA. This suggests that an increase in BMI may lead to a decline in clinical scores.

Obesity is associated with many comorbidities [8-12]. We also encounter these comorbidities in patients requiring arthroplasty. It has been reported that the presence of comorbidities has an effect on weight gain [2]. The data we obtained in our study support the view that the presence and number of comorbidities have an effect on weight gain.

It is known that oral corticosteroids have effects on weight gain [14]. We did not come across an analysis regarding weight change after hip and knee arthroplasty in patients using corticosteroids. In our study, we observed that oral corticosteroid use did not have a significant effect on BMI.

The impact of rehabilitation on weight change is of interest, but uncertainties about rehabilitation quality, intensity, and patient adherence make it hard to determine its precise effect [5]. It has been reported that contrary to expectations, there is no increase in physical activity after arthroplasty [27]. We examined the role of postoperative physiatrist follow-up on patient adherence and compliance. While we expected rehabilitation protocols to improve outcomes, our findings showed increased BMI and decreased Oxford scores in these patients. This paradox may result from the referral of patients with inadequate recovery, affecting the results.

Khow et al. reported that simultaneous bilateral knee arthroplasty had no significant effect on BMI [24]. We are aware that knee arthritis commonly develops bilaterally [28]. Despite joint arthroplasty, the presence of arthritis in the contralateral extremity can limit activity due to pain. Therefore, we deemed it necessary to compare bilateral procedures with unilateral procedures. In our own study, we found that a bilateral simultaneous procedure was associated with a decrease in BMI and an increase in clinical scores.

Limitations of this study include the retrospective design, which may introduce bias, the reliance on self-reported data for physical activity and adherence to rehabilitation protocols, and the exclusion of patients undergoing bariatric surgery, which may limit the generalizability of the findings. Additionally, the study did not control for variations in rehabilitation quality and intensity, potentially impacting the observed relationships between BMI change and functional outcomes.

## Conclusions

This study uniquely examines postoperative weight changes and their impact on functional scores after knee and hip arthroplasty, addressing a multifaceted relationship not previously explored in existing literature. Our results show that BMI changes post-TKA and THA are influenced by various clinical and demographic factors. Increased BMI negatively affects functional outcomes, particularly in hip arthroplasty patients.

Comorbidities significantly influenced weight gain, while oral corticosteroid use had no notable effect on BMI. Patients undergoing bilateral procedures experienced lower BMI increases, with the hip group gaining more weight while the knee group's BMI remained higher throughout the follow-up.

Regarding Oxford scores, comorbidities, corticosteroid use, affected joints, and age did not significantly impact outcomes. Patients under physiotherapist supervision had smaller increases in scores, and although bilateral procedure patients achieved higher scores, simultaneous bilateral procedures did not lead to greater increases.

These findings underscore the importance of weight control and rehabilitation in enhancing postoperative recovery and quality of life.
